# Supplementary material for: Tackling critical parameters in metazoan meta-barcoding experiments: a preliminary study based on coxI DNA barcode
Source: PeerJ. 2018 Jun 13;6:e4845. doi: 10.7717/peerj.4845 (PMC6004112; doi:10.7717/peerj.4845)
Supplement: Supplemental Information 1 — The supplementary materials describe the primer search analysis, which was a necessary step due to the sequencing libraries type. [file peerj-06-4845-s001.docx]

**Tackling critical parameters in metazoan meta-barcoding experiments: a preliminary study based on *coxI* DNA barcode**

**Supplementary Materials**

# Primer search analysis

Due to amplicon concatenation with a ligation reaction and subsequent nebulization steps carried out for sequencing library preparation according to the protocol of ([Calabrese et al. 2013](#_ENREF_1)), different combinations of PCR primers were expected to be found within sequence reads. For that, it was necessary to conduct a pattern search analysis of four possible primer combinations: (a) primer reverse (reverse complement) + primer forward, (b) primer forward (reverse complement) + primer reverse, (c) primer reverse (reverse complement) + primer reverse, (d) primer forward (reverse complement) + primer forward. These analyses were computed by means of Python2.7 script to execute: (i) pairwise global alignment of the four patterns against all sequences using *Needleman–Wunsch* algorithm (EMBOSS package), (ii) comparison between the four alignment scores that considers the highest one as best match, (iii) computation of a modified *Karlin-Altschul* statistic ($E=mne^{-\lambda S}$; where *mn* is the size of the search space, λ=0.27, *S* is the alignment score) that classifies the best scores in three categories: a) good match for E<1e-07, b) bad match for 1e-07<E<1e-05, c) no match for E>1e-05. The two thresholds were chosen looking at the calculated statistic frequency distribution on which the region between the two modes was considered as an ambiguous match. In the case of no match category, sequences were considered ready for downstream analysis without the need of pattern removal, while bad match was discarded from further analyses. Sequences belonging to good match category were spliced up- and down-stream of pattern position. A further match validation using blastn, with E-value < 1e-03 on good match and no match categories, was conducted against the public BOLD: Barcode of Life Data System ([www.barcodinglife.org](http://www.barcodinglife.org)) *coxI* database and checked for correct sequences content (match with *coxI* profile and strand sense). Once this last validation terminates, the script outputs four trimming lists indicating pattern position when present. Sequence reads trimming has generated for each sample two separate data sets at 5’ and 3’ *coxI* barcode, used in the subsequent denoising step.

## References

Calabrese C, Mangiulli M, Manzari C, Paluscio AM, Caratozzolo MF, Marzano F, Kurelac I, D'Erchia AM, D'Elia D, Licciulli F, Liuni S, Picardi E, Attimonelli M, Gasparre G, Porcelli AM, Pesole G, Sbisa E, and Tullo A. 2013. A platform independent RNA-Seq protocol for the detection of transcriptome complexity. *BMC Genomics* 14:855. 10.1186/1471-2164-14-855
